# Systemic Lupus Erythematosus and Lupus Nephritis Presenting as Severe Constitutional Symptoms

**DOI:** 10.7759/cureus.56670

**Published:** 2024-03-21

**Authors:** Ton La, Sahifah Ansari, Ethan Ha, Matthew Novakovic

**Affiliations:** 1 Internal Medicine, Baylor College of Medicine, Houston, USA; 2 Internal Medicine, University of Texas Medical Branch, Galveston, USA

**Keywords:** systemic lupus erythematosus, lupus nephritis, facial cellulitis, pericardial effusion, anemia of chronic disease

## Abstract

Systemic lupus erythematosus (SLE) is a remitting-relapsing systemic autoantibody and immune complex disease with a similar clinical presentation to that of malignancy and infection. The authors report a case of newly diagnosed SLE and lupus nephritis in a 48-year-old woman with constitutional symptoms and unintentional weight loss. Her presentation was further complicated by pericardial effusion and methicillin-resistant Staphylococcus aureus (MRSA) facial cellulitis and bacteremia. In the context of nonspecific symptoms and a wide initial differential diagnosis, the early consideration of rheumatologic etiologies and the involvement of consultant services led to appropriate diagnostic testing and a timely diagnosis.

## Introduction

Systemic lupus erythematosus (SLE) is a remitting and relapsing systemic autoantibody and immune complex disease that can present with a similar clinical picture to that of malignancy (lymphoma and leukemia) and infection (tuberculosis, HIV/AIDS, and bacterial endocarditis) [1]. To classify SLE, the 2019 European League Against Rheumatism (EULAR)/American College of Rheumatology (ACR) criteria include a positive antinuclear antibody (ANA) at least once as an obligatory entry criterion. Then, additive criteria grouped in seven clinical (constitutional, hematologic, neuropsychiatric, mucocutaneous, serosal, musculoskeletal, and renal) and three immunologic (antiphospholipid antibodies, complement proteins, and SLE-specific antibodies) domains are weighted from two to 10 [2]. Patients accumulating ≥10 points are classified as having SLE for the purpose of research studies, but patients with fewer points can still be clinically diagnosed with SLE based on the overall picture of their disease [2]. SLE patients may present with constitutional symptoms such as fatigue, weight loss, and fever. Lupus nephritis is the most life-threatening complication that affects about 30% of SLE patients. SLE can also affect multiple organ systems including skin, musculoskeletal, cardiopulmonary, and gastrointestinal. Depending on the severity of the disease, the treatment of SLE includes corticosteroids and immunosuppressive drugs. We report a case of newly diagnosed systemic lupus erythematosus and lupus nephritis complicated by pericardial effusion and methicillin-resistant Staphylococcus aureus (MRSA) facial cellulitis and bacteremia. [1]

This article was previously presented as a poster at the 2023 Annual Rheumatology Nurses Society Conference on August 3, 2023.

## Case presentation

A 48-year-old woman with a past medical history of rheumatoid arthritis (RA) diagnosed in 2019 at an outside facility presented to the emergency department from clinic for a hemoglobin of 7.8 g/dL. She also endorsed fatigue, weakness, shortness of breath, generalized abdominal pain, and unintentional weight loss of 100 pounds in the last two to three months. Her fatigue started three years ago before she developed the additional symptoms in the last three months. She left her job as a janitor due to the severity of her symptoms, specifically sharp, intermittent abdominal pain, which prompted her to visit her primary care physician.

On presentation, her vital signs were normal. The physical exam was notable for non-blanchable petechiae on the left medial foot, trace proximal interphalangeal joint synovitis, and trace wrist effusion. The abdomen was nondistended without rebound or guarding, and the digital rectal exam revealed no blood. Labs were significant for lymphopenia (WBC was 3.5 103/uL, normal 4.5-11.0 103/uL; absolute lymphocyte count 0.41x10^3/uL, normal 1.18-3.74x10^3/uL) and normocytic anemia (Hgb was 7.3 g/dL, normal 12.0-16.0 g/dL; mean corpuscular volume (MCV) was 81.1fL, normal 82.0-92.0 fL) without any signs of bleeding. Her ferritin was 608.7 ng/mL (normal 11.0-306.8 ng/mL), total iron-binding capacity (TIBC) was 136 ug/dL (normal 250-450 ug/dL), and iron saturation was 15% (normal 15-55%), which were consistent with anemia of chronic disease. Direct antiglobulin test was positive, though the antibody was non-specific, and other hemolysis labs including LDH, haptoglobin, and reticulocyte count were all unremarkable. Peripheral blood smear revealed marked normocytic normochromic anemia with moderate anisopoikilocytosis including dacrocytes and elliptocytes.

Autoimmune labs were overwhelmingly positive with an ANA ≥ 1:640, low complements (C3 of 29.4 mg/dL, normal 87.0-200.0 mg/dL; C4 of <15 mg/dL, normal 19.0-52.0 mg/dL), positive Anti-DsDNA 1:1280, positive Sjogren’s Anti-SS-A/Anti-SS-B of >8.0 (normal 0.0-0.9 AI), and positive anticardiolipin IgM of 24 MPL U/mL (normal 0-12 MPL U/mL). She met the 2019 EULAR/ACR classification criteria for SLE with 38 points, well above the minimum of 10 points (Table 1 and Table 2). Rheumatology was consulted, and their plan was to give pulse dose steroids (methylprednisolone sodium succinate 1g IV daily for three doses) before transitioning to oral prednisone 60mg daily and mycophenolate mofetil 500mg twice daily. However, the patient developed MRSA facial cellulitis and uncomplicated bacteremia during her hospital course after the pulse dose steroids, so her maintenance prednisone was decreased to 20mg daily and mycophenolate mofetil was held until the infection resolved. The patient's infection resolved with IV vancomycin.

**Table 1:** 2019 European League Against Rheumatism (EULAR)/American College of Rheumatology (ACR) classification criteria for systemic lupus erythematosus (SLE) with patient score, clinical domains *Positive finding but additional criteria within the same domain are not counted toward the total score. A total score of ≥10 in both the clinical and immunological domains with at least one clinical criterion is required to classify SLE.

Clinical domains and criteria (weight)	Patient score
Constitutional	
Fever (2)	2
Hematologic	
Leukopenia (3)	3
Thrombocytopenia (4)	0
Autoimmune hemolysis (4)	0
Neuropsychiatric	
Delirium (2)	0
Psychosis (3)	0
Seizure (5)	0
Mucocutaneous	
Non-scarring alopecia or oral ulcers (2)	0
Subacute cutaneous lupus or discoid lupus (4)	0
Acute cutaneous (6)	0
Serosal	
Pleural or pericardial effusion (5)	5
Acute pericarditis (6)	0
Renal	
Proteinuria >0.5/24h (4)*	0
Class II or V lupus nephritis (8)	0
Class II or IV lupus nephritis (10)	10
Musculoskeletal	
Joint involvement	6
Clinical domains criteria patient total	26

**Table 2:** 2019 European League Against Rheumatism (EULAR)/American College of Rheumatology (ACR) classification criteria for systemic lupus erythematosus (SLE) with patient score, immunologic domains *Positive finding but additional criteria within the same domain are not counted. A total score of ≥10 in both the clinical and immunological domains with at least one clinical criterion is required to classify SLE.

Immunologic domains and criteria (weight)	Patient score
Antiphospholipid antibodies	
Anti-cardiolipin antibodies OR	
Anti-B2GP1 antibodies OR	
Lupus anticoagulant (2)	2
Complement levels	
Low C3 or low C4 (3)*	0
Low C3 and low C4 (4)	4
SLE-specific antibodies	
Anti-dsDNA OR anti-Smith (6)	6
Immunologic domains criteria patient total	12

**Figure 1:**
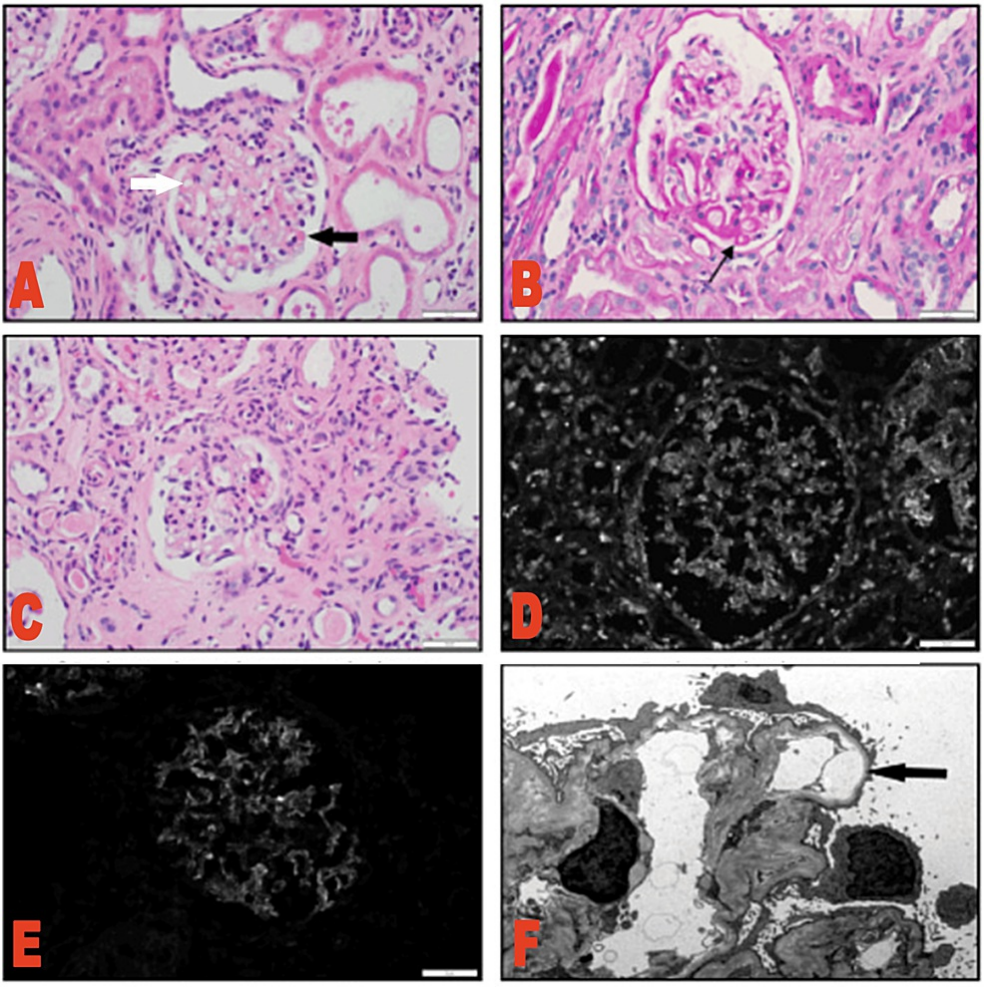
Kidney biopsy photomicrographs (A) H&E: Glomerulus with segmental wire loops (white arrow) and hyaline thrombi (black arrow). (B) Periodic acid-Schiff (PAS): Glomerulus with segmental wire loops (arrow). (C) H&E: Glomerulus with segmental sclerosis. (D) IgG: Glomerular deposits. (E) C1q: Glomerular deposits. (F) Electron microscopy (EM): Segmental subendothelial rarefaction (arrow).

She was also found to have proteinuria of 0.44 g/L (normal <0.19 g/L), hematuria with 88 RBC/HPF (normal 0-4 RBC/HPF), and pyuria with 20 WBC/HPF (normal 0-5 WBC/HPF). Rheumatology recommended a renal biopsy based on the patient's high EULAR/ACR score and abnormal urinalysis. Nephrology was consulted, and a renal biopsy revealed class IV lupus nephritis (Figure 1) and mild thrombotic microangiopathy (TMA). The patient also had elevated antiphospholipid antibodies, but she did not meet clinical criteria for antiphospholipid syndrome (APS) per the 2019 EULAR/ACR guidelines. The gastrointestinal (GI) department was consulted to perform an esophagogastroduodenoscopy (EGD) and colonoscopy, which did not reveal a cause for the patient’s anemia or weight loss.

**Figure 2:**
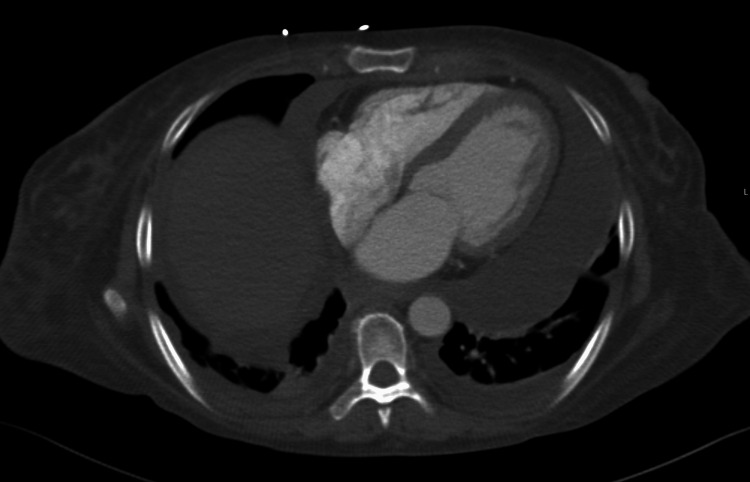
CT image of pericardial and pleural effusions

Transthoracic echocardiogram (TTE) showed no evidence of endocarditis; however, the patient was found to have right ventricle diastolic collapse compatible with a hemodynamically significant pericardial effusion without clinical signs of tamponade (Figure 2). Serial TTEs showed stable effusion size with improvement in diastolic collapse. There was no urgent indication for pericardiocentesis given the patient’s hemodynamic stability. The patient scored a 20 on the Systemic Lupus Erythematosus Disease Activity Index 2000 (SLEDAI-2K) for the abnormal lab findings and disease manifestations of arthritis and pericardial effusion, representing active disease.

## Discussion

Patients with SLE can lose weight due to disease activity and its major manifestations, but it is important to evaluate for other causes of weight loss like infection and malignancy by EGD and colonoscopy. The prompt initiation of interdisciplinary care by involving GI, rheumatology, and nephrology consultant teams was a key factor in obtaining a timely and accurate diagnosis in this case.

The patient’s positive direct Coombs test led to further workup for hemolysis which was negative. A positive direct Coombs test in the absence of hemolytic anemia in SLE patients may indicate high disease activity and poor renal response in SLE [3]. In one study, 5.8% of 182 patients with SLE who had a positive direct Coombs test in the absence of hemolytic anemia had higher SLE disease activity index scores (p < 0.01) and lower complete renal response rates (p = 0.03) over one year after induction therapy for lupus nephritis compared to those who had a negative direct Coombs test without hemolytic anemia [3]. However, this study was limited because it was a single-center retrospective study with a small and ethnically homogenous sample population. A multicenter, prospective study that includes patients from multiple races is warranted; however, it is prudent to consider direct Coombs test results as a factor when assessing illness severity. The TMA found on the patient's renal biopsy is unlikely to represent a thrombotic event that would be attributable to APS. She has no prior history of clotting, pregnancy, or miscarriage, and an official diagnosis of APS requires clinical manifestations such as recurrent venous or arterial thrombosis with or without pregnancy loss [4].

SLE often involves the pericardium leading to common cardiac complications including pericarditis, pericardial effusion, and rarely cardiac tamponade secondary to pericardial effusion as the presenting symptoms of SLE [5]. A large pericardial effusion, as seen in this patient, is rare and is associated with lupus nephritis, Libman-Sacks endocarditis, and myocardial dysfunction [6]. In one prospective study, treatment of patients with SLE using oral corticosteroids and complete drainage of the large pericardial effusions was associated with good cardiac outcomes [6]. In a systematic review and meta-analysis, SLE was found to be associated with significant structural and functional cardiac abnormalities seen in TTE [7]. This study suggests that echocardiographic assessments should be part of routine examinations for patients with SLE [7]. In a symptomatic patient, as seen in this case, early assessment becomes even more warranted.

Of note, the patient’s significant weight loss persisted over many months before she sought care. The lack of insurance and loss of income after she had to leave her job because of her illness contributed to her delayed diagnosis and treatment. These challenges are not unique to this patient and have been experienced by many others throughout the ongoing COVID-19 pandemic. Prompt identification of social risk factors through established screening of the patient's demographics and knowledge of ongoing barriers to care within and outside of a patient's control can provide insight into the severity of disease activity at presentation and prevent delays in receiving further care.

## Conclusions

This patient’s age, weight loss, delayed presentation, and cardiac and infectious complications were a unique presentation of SLE. Patients experiencing barriers to care may have their initial presentation occur at a severe disease state. Constitutional symptoms are non-specific and require a complete workup after ruling out emergent or malignant causes. Early involvement of consultant care led to the correct diagnosis using appropriate diagnostic testing and established EULAR/ACR classification criteria.
